# Cardiac development demystified by use of the HDBR atlas

**DOI:** 10.1111/joa.14066

**Published:** 2024-05-23

**Authors:** Robert H. Anderson, Janet Kerwin, Wouter H. Lamers, Jill P. J. M. Hikspoors, Timothy J. Mohun, Bill Chaudhry, Steven Lisgo, Deborah J. Henderson

**Affiliations:** ^1^ Biosciences Institute Newcastle University Newcastle upon Tyne UK; ^2^ Human Developmental Biology Resource, Biosciences Institute Newcastle University Newcastle upon Tyne UK; ^3^ Department of Anatomy and Embryology Maastricht University Maastricht The Netherlands; ^4^ Crick Institute London UK

**Keywords:** cardiac development, Carnegie stages, HDBR, histology atlas, human embryo

## Abstract

Much has been learned over the last half century regarding the molecular and genetic changes that take place during cardiac development. As yet, however, these advances have not been translated into knowledge regarding the marked changes that take place in the anatomical arrangements of the different cardiac components. As such, therefore, many aspects of cardiac development are still described on the basis of speculation rather than evidence. In this review, we show how controversial aspects of development can readily be arbitrated by the interested spectator by taking advantage of the material now gathered together in the Human Developmental Biology Resource; HDBR. We use the material to demonstrate the changes taking place during the formation of the ventricular loop, the expansion of the atrioventricular canal, the incorporation of the systemic venous sinus, the formation of the pulmonary vein, the process of atrial septation, the remodelling of the pharyngeal arches, the major changes occurring during formation of the outflow tract, the closure of the embryonic interventricular communication, and the formation of the ventricular walls. We suggest that access to the resource makes it possible for the interested observer to arbitrate, for themselves, the ongoing controversies that continue to plague the understanding of cardiac development.

## Graphical abstract

3D reconstructions, histological, and HREM sections of CS13–14 human hearts.

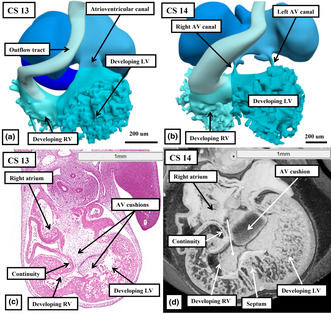

## 1 INTRODUCTION

We have learned much over the past 50 years regarding cardiac development, largely from studies in animal models. These have mainly focussed on the investigation of developing mouse hearts, both normal and abnormal. More recently, the dynamic changes in shape of the developing heart have been elucidated by the availability of techniques that permit three‐dimensional reconstruction of the embryo, such as high‐resolution episcopic microscopy (Mohun & Weninger, 2012). This rapid increase in our understanding of the mouse heart, however, has not been matched by a similar understanding of the developing human heart. This is, in part, because of the limited availability of human embryos for study, but also because mouse and human hearts, although similar, are not identical. As a consequence, many textbooks used by students learning cardiac anatomy, or those used to aid the understanding of congenital cardiac malformations, are significantly out of date when it comes to our understanding of human cardiac development. Some recent accounts of human cardiac development are now available (Buijtendijk et al., 2020), but they do not provide the detail necessary to understand all the malformations. Many of these detailed issues were clarified by the provision of interactive PDFs permitting three‐dimensional reconstructions of serially sectioned human embryos, based on the Carnegie collection (Hikspoors et al., 2022). When this is complemented with the histological data now available on the website of the Human Developmental Biology Resource (hdbr.org), major leaps forward can be achieved. The site now contains annotated datasets of recently collected human embryos, in multiple orientations, permitting the reader to scroll through serial histological sections. Along with annotated datasets, they show the key changes that take place during the stages of cardiac development from 3 weeks post‐conception to the end of the embryonic period. In this review, we have used selected images from the website, including some prepared using episcopic microscopy (Mohun & Weninger, 2012), to demonstrate these changes. We have combined them, where appropriate, with images prepared from the interactive PDFs published previously (Hikspoors et al., 2022). These files are available within the HDBR Atlas. Together, they illustrate the process of development of the heart from a solitary tube to the definitive four‐chambered organ.

## 2 THE HUMAN DEVELOPMENTAL BIOLOGY RESOURCE

The resource is basically a tissue bank. It was established so that biological samples could be collected and stored, with the permission of the donor, so that they could then be used by researchers in need of human embryonic and foetal material for their studies. The material has been collected and banked since 1999, funded throughout this period by the Medical Research Council of the United Kingdom and the Wellcome Trust. The resource has been licensed as a tissue bank at each of its sites, which are located at the Bioscience Institute of Newcastle University, and the Institute of Child Health, University College London. Since its inception, the resource has supported over 600 research projects in many countries.

**FIGURE 1:**
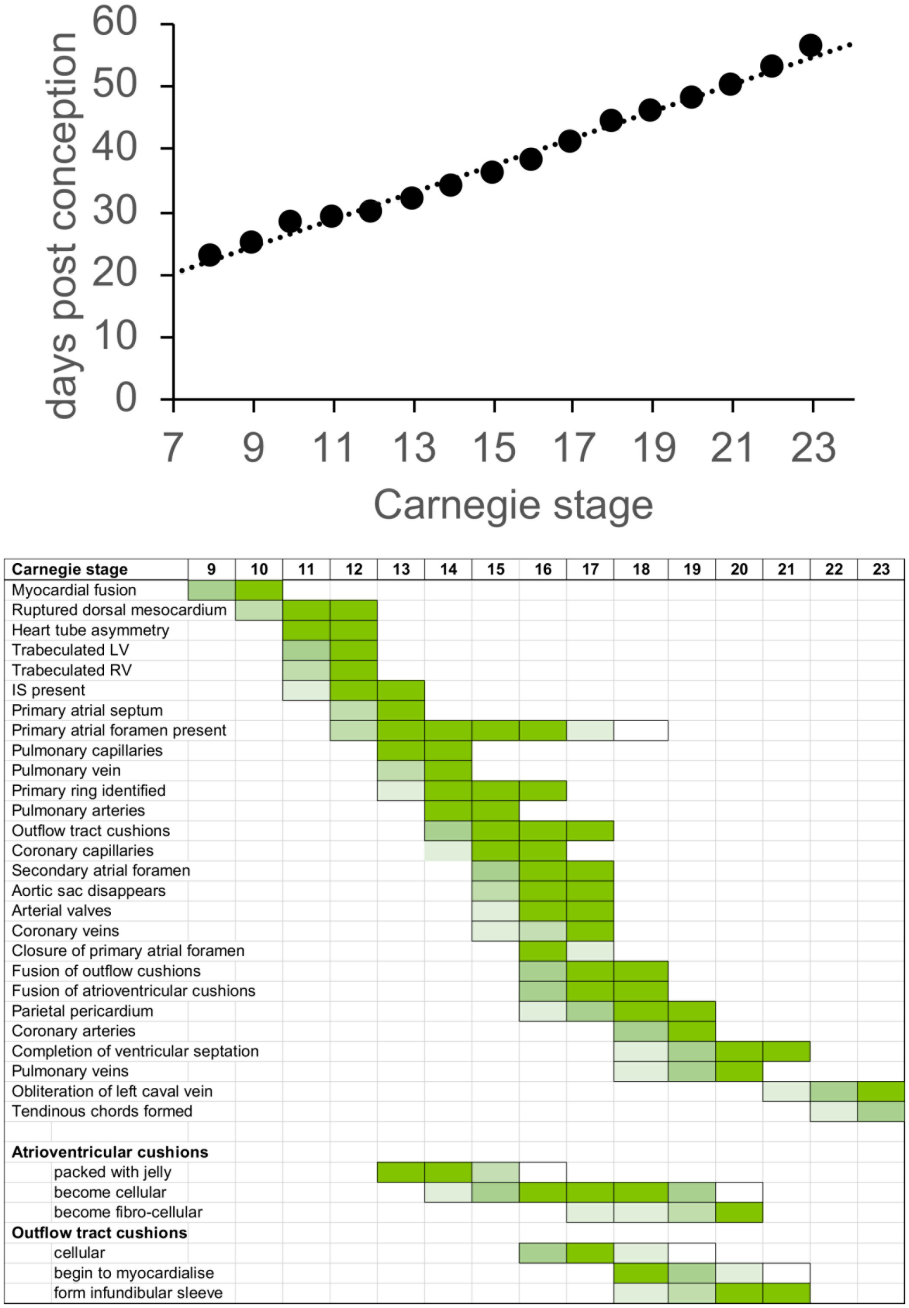
The upper panel shows the approximate stages of cardiac development related to the Carnegie staging as modified by O'Rahilly and Müller (2010). The lower panel shows the approximate stages of appearance of the various cardiac components, with the density of colouring showing the approximations in terms of timing. IS, interventricular septum; LV, left ventricle; RV, right ventricle. The primary ring refers to the ring of cardiomyocytes that surrounds the primary interventricular foramen.

Over and above the provision of human tissues for researchers, it has proved possible to develop a significant Atlas of Human Development (hdbratlas.org). The atlas provides high resolution histological sections, in three orthogonal planes, of whole embryos covering the first 8 weeks of development. The staging of the embryos has been based on external anatomical features as described by the Carnegie classifications (O'Rahilly & Müller, 2010), although with some minor modifications (see https://hdbratlas.org/staging‐criteria/carnegie‐staging.html). Each of the embryos in the atlas was in perfect condition on collection, was typical for its stage of development and was karyotypically normal, with no evidence of structural abnormalities. Relying solely upon external features to stage an embryo, including the numbers of somite pairs and aspects of limb and eye development, however, is able to provide only an approximation of the developmental maturity of the embryo as a whole. The complex and dynamic changes taking place within specific organs do not necessarily precisely match changes observed at the gross external morphological level. For example, there can be some degree of asynchrony between the heart and the development of the external features of the embryo (Figure 1). When considered in the context of cardiac development especially, the specific timings are at best approximate. After 8 weeks of gestation, the primitive organism is described as a foetus, and staging is based on post‐conceptional weeks. Data are also available for these stages, although these later stages of development are not the focus of this review.

Already the website provides access to movies, interactive models, and dissections of human embryonic and foetal hearts prepared by the team in Newcastle, and the group of Dr Heather Etchevers, Marseille Medical Genetics, France. It is also possible to scroll through three‐dimensional datasets prepared using episcopic microscopy for six stages of development, as prepared by Dr Tim Mohun. It is the histological sections, nonetheless, that are of greatest value to those who wish to assess for themselves the rapid changes that take place during cardiac development. New material is being loaded into the website all the time as more embryos are prepared at the Newcastle site. It is selected sections from these datasets, including episcopic images, and combined where appropriate with reconstructions of the interactive files created by Hikspoors and colleagues, which we have used to illustrate the key morphological processes that take place during remodelling of the human heart tube to produce the four‐chambered organ. Methodological information is available at https://hdbratlas.org/protocols.html and in Mohun and Weninger (2012) and Hikspoors et al. (2022). We have listed the specific histological sections used to make our figures, along with the details required to locate them on the web, in Table 1. Although not included here, foetal hearts have also been collected and some images of the foetal heart are already available within the atlas, with the goal to make these datasets available in the coming year.

**TABLE 1:** Weblinks to images used in the figures.

Figure	Stage	HDBR atlas weblink
2b	CS10	\https://histology.hdbratlas.org/?config=/HDBR/config.json&model=CS10_A135T&image=7
2d	CS10	https://histology.hdbratlas.org/?config=/HDBR/config.json&model=CS10_A135T&image=8
4a	CS12	https://hdbratlas.org/organ‐systems/cardiovascular‐system/heart/heart‐sections/CS12/11638_16.html
4b	CS12	https://hdbratlas.org/organ‐systems/cardiovascular‐system/heart/heart‐sections/CS12/11638_24.html
4c	CS12	https://hdbratlas.org/organ‐systems/cardiovascular‐system/heart/heart‐sections/CS12/11638_28.html
4d	CS12	https://hdbratlas.org/organ‐systems/cardiovascular‐system/heart/heart‐sections/CS12/11638_32.html
5c	CS13	https://hdbratlas.org/organ‐systems/cardiovascular‐system/heart/heart‐sections/CS13/11559_63.html
6d	CS13	https://hdbratlas.org/organ‐systems/cardiovascular‐system/heart/heart‐sections/CS13/11559_59.html
6e	CS14	https://hdbratlas.org/organ‐systems/cardiovascular‐system/heart/heart‐sections/CS14/174_088.html
7a	CS15	https://hdbratlas.org/organ‐systems/cardiovascular‐system/heart/heart‐sections/CS15/11847_119.html
7b	CS16	https://hdbratlas.org/organ‐systems/cardiovascular‐system/heart/heart‐sections/CS16/CS16_N2170_97.html
7c	CS18	https://hdbratlas.org/organ‐systems/cardiovascular‐system/heart/heart‐sections/CS18/482_209.html
7d	CS21	https://hdbratlas.org/organ‐systems/cardiovascular‐system/heart/heart‐sections/CS21/CS21_N641_291.html
8b	CS15	https://hdbratlas.org/organ‐systems/cardiovascular‐system/heart/heart‐sections/CS15/11847_113.html
8c	CS16	https://hdbratlas.org/organ‐systems/cardiovascular‐system/heart/heart‐sections/CS16/CS16_N2170_93.html
8d	CS18	https://hdbratlas.org/organ‐systems/cardiovascular‐system/heart/heart‐sections/CS18/482_205.html
9a	CS14	https://histology.hdbratlas.org/?config=/HDBR/config.json&model=CS14_1930F&image=28
9b	CS17	https://hdbratlas.org/histology/pas/cs17/CS17_pas_150.html
9c	CS18	https://histology.hdbratlas.org/?config=/HDBR/config.json&model=CS18_N998F&image=16
9d	CS21	https://histology.hdbratlas.org/?config=/HDBR/config.json&model=CS21_13436F&image=21
13a	CS16	https://hdbratlas.org/organ‐systems/cardiovascular‐system/heart/heart‐sections/CS16/CS16_N2170_73.html
13b	CS17	https://hdbratlas.org/organ‐systems/cardiovascular‐system/heart/heart‐sections/CS17/365_691.html
13c	CS18	https://hdbratlas.org/organ‐systems/cardiovascular‐system/heart/heart‐sections/CS18/482_175.html
13d	CS21	https://hdbratlas.org/organ‐systems/cardiovascular‐system/heart/heart‐sections/CS21/CS21_N641_261.html
14a	CS17	https://hdbratlas.org/organ‐systems/cardiovascular‐system/heart/heart‐sections/CS17/365_761.html
14b	CS18	https://hdbratlas.org/organ‐systems/cardiovascular‐system/heart/heart‐sections/CS18/482_189.html
14c	CS19	https://hdbratlas.org/organ‐systems/cardiovascular‐system/heart/heart‐sections/CS19/15227_13.html
14d	CS20	https://hdbratlas.org/organ‐systems/cardiovascular‐system/heart/heart‐sections/CS20/446_141.html
15a	CS17	https://hdbratlas.org/organ‐systems/cardiovascular‐system/heart/heart‐sections/CS17/365_781.html
15b	CS19	https://hdbratlas.org/organ‐systems/cardiovascular‐system/heart/heart‐sections/CS19/15227_15.html
15c	CS20	https://hdbratlas.org/organ‐systems/cardiovascular‐system/heart/heart‐sections/CS20/446_143.html
15d	CS22	https://hdbratlas.org/organ‐systems/cardiovascular‐system/heart/heart‐sections/CS22/11949_17.html
16a	CS18	https://hdbratlas.org/organ‐systems/cardiovascular‐system/heart/heart‐sections/CS18/482_193.html
16b	CS21	https://hdbratlas.org/organ‐systems/cardiovascular‐system/heart/heart‐sections/CS21/CS21_N641_275.html
16c	CS23	https://hdbratlas.org/organ‐systems/cardiovascular‐system/heart/heart‐sections/CS23/300_13.html
16d	CS23	https://hdbratlas.org/organ‐systems/cardiovascular‐system/heart/heart‐sections/CS23/300_18.html

## 3 FORMATION OF THE VENTRICULAR LOOP

**FIGURE 2:**
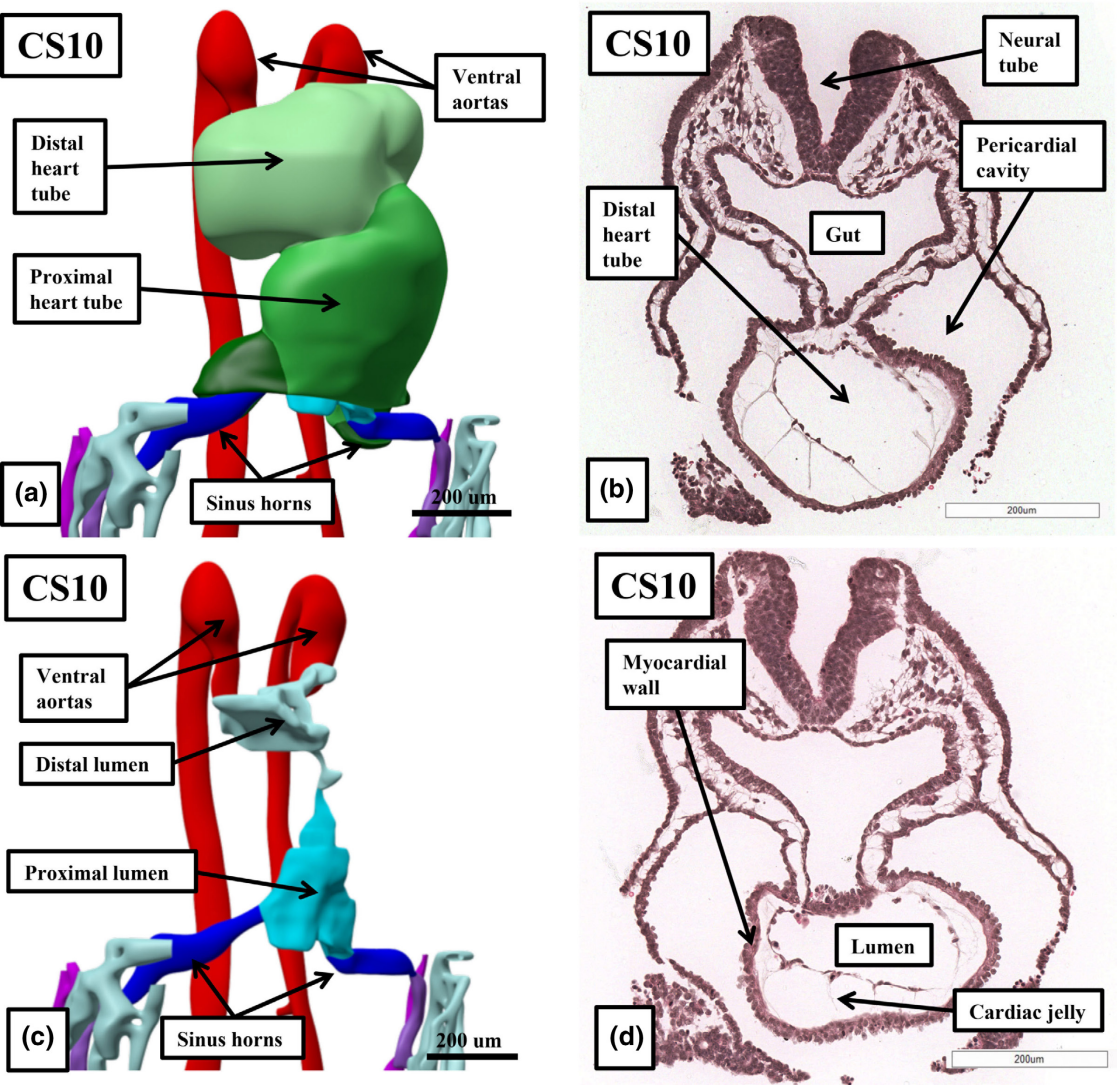
Panels a and c are created from an interactive PDF prepared using a human embryo at Carnegie stage 10. The sections shown in panels b and d are from a dataset contained in the HDBR atlas. The reconstructions show the linear arrangement of the heart tube at this stage, with the sections showing the myocardial mantle that clothes the inlet and outlet components.

**FIGURE 3:**
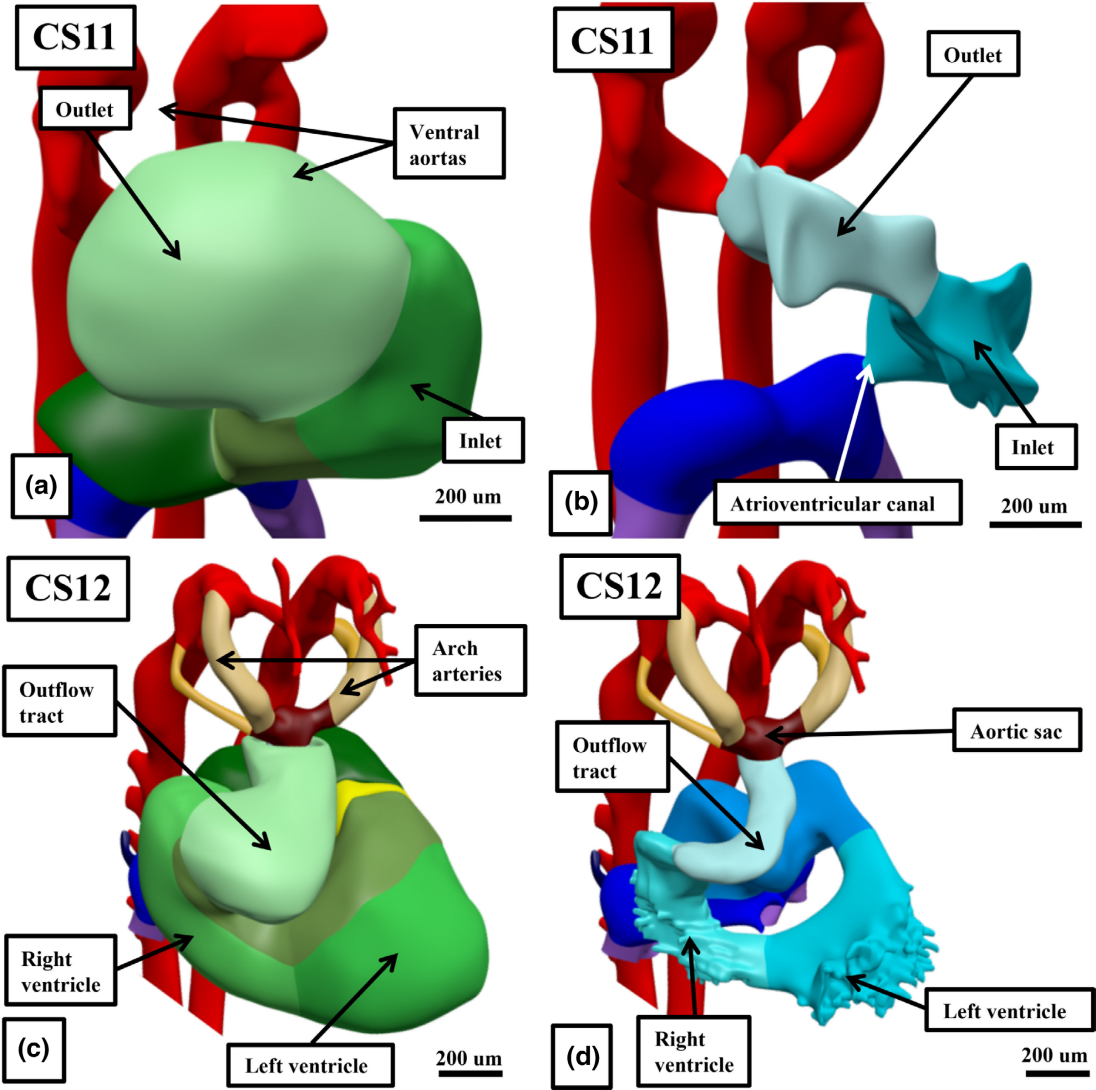
The panels, again prepared from interactive PDFs but reconstructed from human embryos at Carnegie stage 11 (a, b) and 12 (c, d), show how addition of new material from the second heart field leads initially to the production of the ventricular loop (a, b), with the development of trabeculations from the outer curvature (lower panels) producing the apical components of the developing right and left ventricles.

**FIGURE 4:**
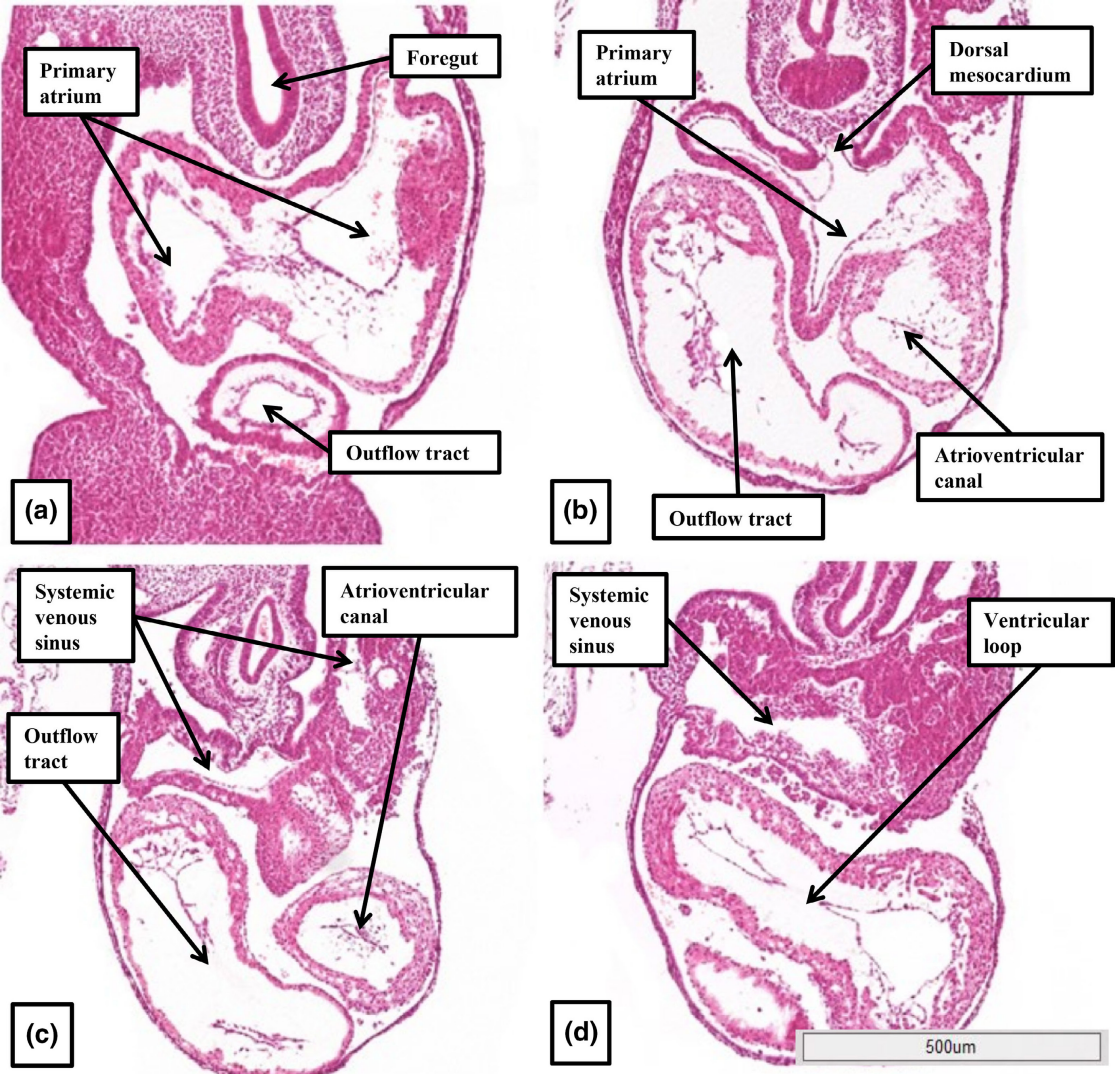
The images show serial sections taken from the human embryo 11,638, dated at Carnegie stage 12, and annotated in the Atlas. Panel a shows a cross section through the atrial component of the heart tube, with the outflow tract seen ventrally. Panel b shows the atrioventricular canal and the top of the ventricular loop. The dorsal mesocardium is also seen—connecting the atrial component to the pharyngeal mesenchyme. Panel c cuts through the outflow tract and the atrioventricular canal, with panel d showing the body of the ventricular loop, which remains filled with cardiac jelly.

At Carnegie stage 10, which is approximately 4 weeks after conception, the heart tube has a solitary cavity (Figure 2). Removal of the walls of the chambers within the interactive files shows the cavity to have inlet and outlet components (Figure 2a,c). The sections show the lumen to be surrounded by a thin endocardial layer, cardiac jelly, and encased intrapericardially by a myocardial mantle (Figure 2b,d). At the arterial pole, ventral aortas extend in bilateral fashion to become the dorsal aortas, whilst at the venous pole, the vitelline and umbilical venous plexuses merge, again in bilateral fashion, to open into the inlet of the heart tube as the sinus horns. During Carnegie stages 11 and 12, concomitant with ongoing migration of tissues from the second heart fields at the venous and arterial poles (Kelly et al., 2014), there has been formation of the cardiac loop (Figures 2 and 3). The contours of the loop are best appreciated when viewing a reconstruction (Figure 3). At Carnegie stage 11, the ventricular loop remains a solitary tube, with a smooth‐walled lumen. The addition of new material at the venous pole has produced the primordium of the developing atrial component, with the cardinal venous plexuses, having now developed within the body of the embryo, open bilaterally into the systemic venous inputs. At the arterial pole, the new material has formed the outflow tract, which extends from the outlet of the developing right ventricle to the margins of the pericardial cavity. By Carnegie stage 12, trabeculations are extending from the outer curvature of the ventricular loop. The formation of the trabeculations coincides with the expansion of the ventricular chambers from the developing ventricular loop, a process described by Christoffels and colleagues as “ballooning,” with comparable “ballooning” from the atrial component of the heart tube subsequently producing the atrial appendages (Christoffels et al., 2000). The appearance of the trabeculations within the inlet of the ventricular loop provides the primordium of the developing left ventricle, with the primordium of the right ventricle ballooning from the outlet component of the loop. These processes, and the communications between the ventricular loop, the developing atrial component, and the developing outflow tract can all be appreciated by scrolling through the dataset in the HDBR Atlas prepared from a human embryo at Carnegie stage 12 (Figure 4).

## 4 EXPANSION OF THE ATRIOVENTRICULAR CANAL

**FIGURE 5:**
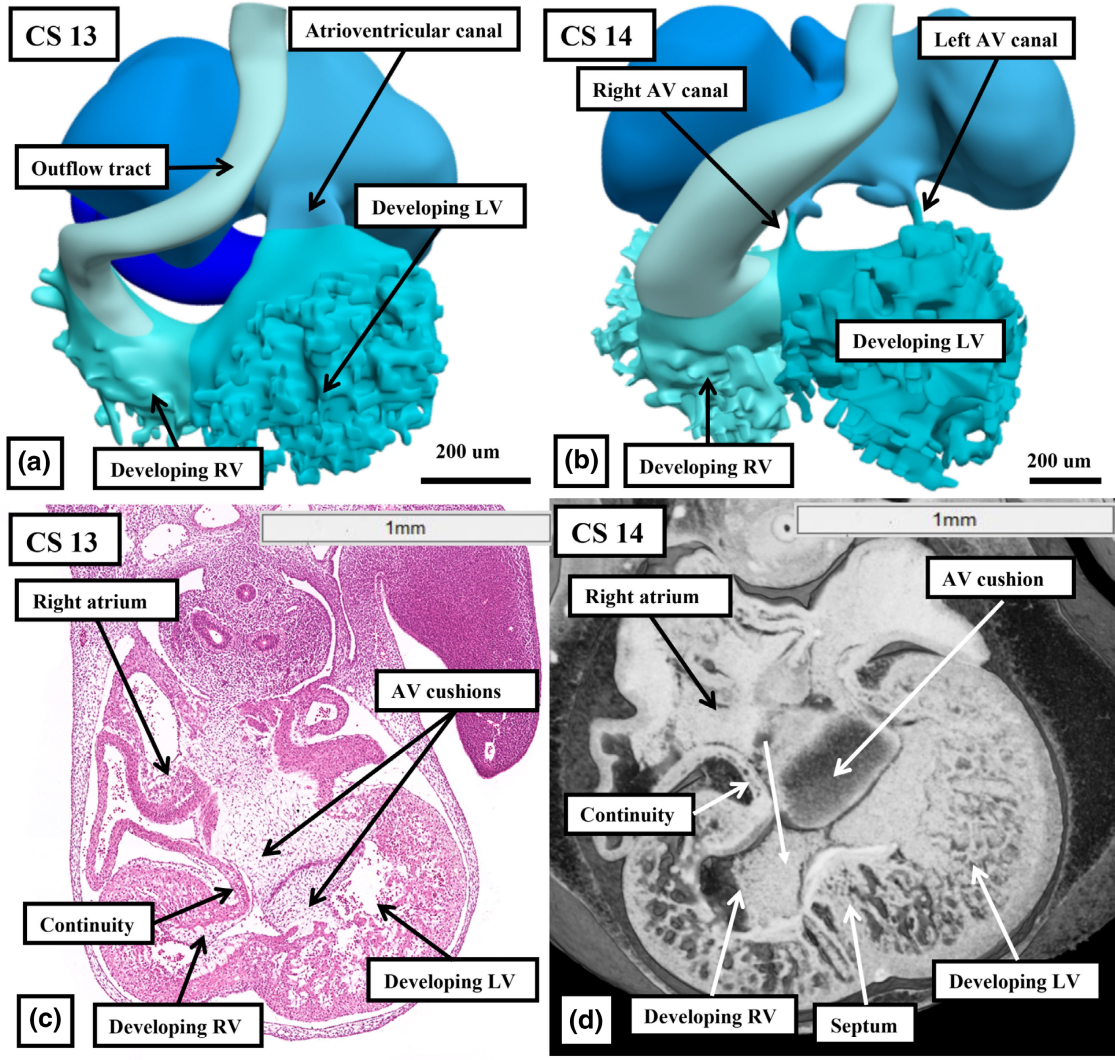
Panel a shows a reconstruction prepared from an interactive PDF from a human embryo at Carnegie stage 13, with panel c showing a section taken from the annotated dataset of human embryo 11,559, also staged at Carnegie stage 13. The atrioventricular canal at this stage is supported exclusively above the cavity of the developing left ventricle, but as seen in panel c, the walls of the developing right atrium and ventricle are already in continuity in the right border of the atrioventricular canal. Panel b shows an interactive reconstruction revealing the connections of the cavities at Carnegie stage 14, with panel d showing a image prepared from an episcopic dataset also from a human embryo staged at Carnegie stage 14. The cavity of the right atrium is now in direct continuity with that of the right ventricle (white arrow). The reconstruction in panel b shows that the outflow tract remains supported exclusively above the cavity of the right ventricle.

Towards the end of the twentieth century, a further topic of contention was the fashion in which the developing right ventricle acquired its inlet component. Subsequent to the formation of the ventricular loop, and at Carnegie stage 13, the atrioventricular canal empties exclusively into the developing left ventricle, whilst the outflow tract is supported exclusively above the cavity of the developing right ventricle (Figure 5a,c). By Carnegie stage 14, at the beginning of the fifth week after conception, the right ventricle has acquired its inlet component simply by expansion of the rightward margin of the atrioventricular canal (Figure 5b,d). Already at this stage of formation of the ventricular loop, the wall of the right atrium is in continuity with the roof of the developing right ventricle in the cranial margin of the primary interventricular communication (Figure 5c). Expansion of the canal, now filled by the atrioventricular cushions, produces direct continuity between the cavities of the right atrium and the right ventricle (Figure 5d). With this change, the dorsal margin of the interventricular communication itself has become the boundary of the developing right atrioventricular junction. It is the persisting part of the primary interventricular foramen, which can now be designated as a secondary communication, that now provides the outlet for the developing left ventricle. This is because the entirety of the outflow tract remains supported above the cavity of the developing right ventricle.

## 5 FORMATION OF THE SYSTEMIC VENOUS SINUS

**FIGURE 6:**
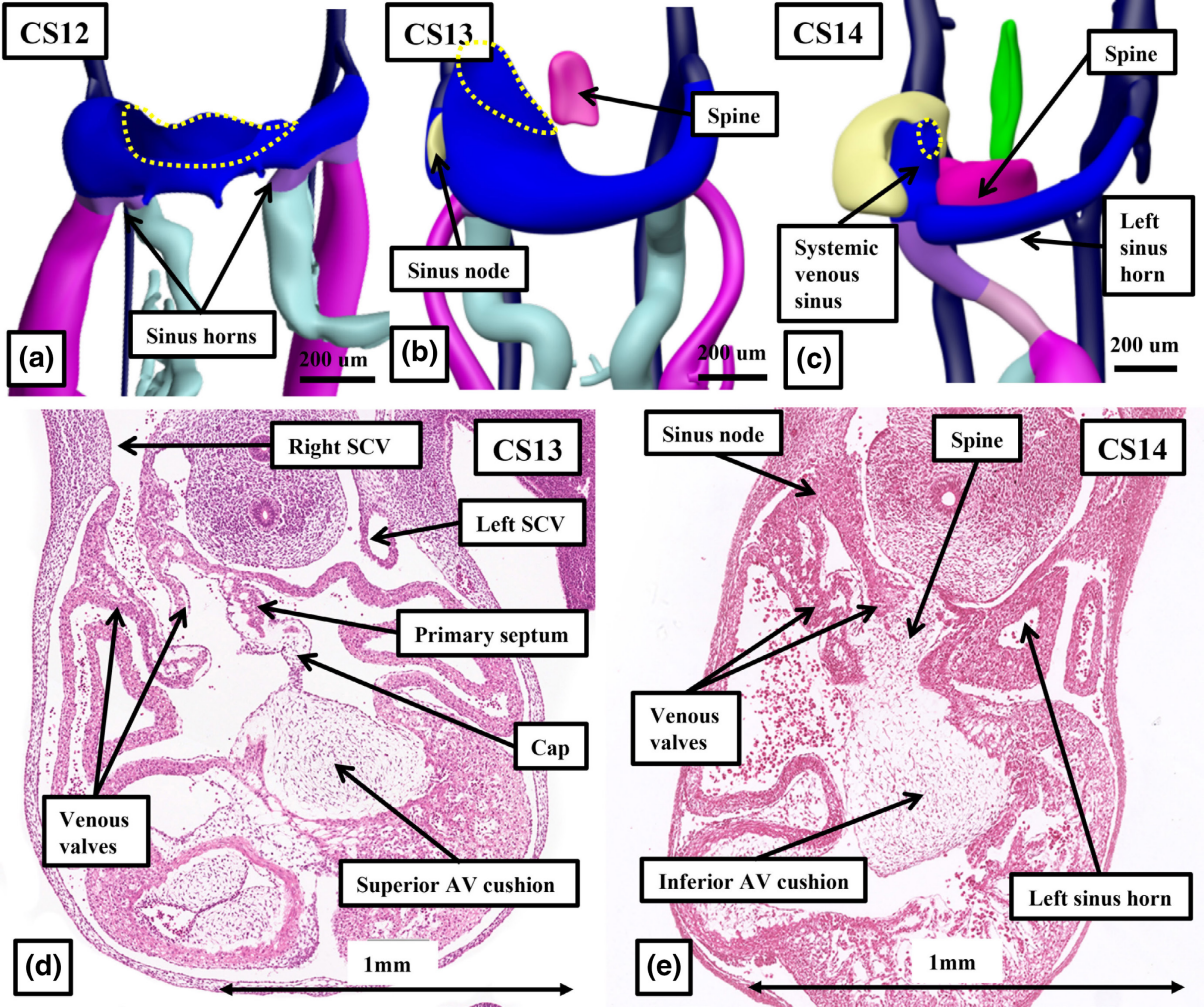
The reconstructions from interactive PDFs, along with sections taken from the annotated datasets in the HDBR Atlas, show the changes occurring as the systemic venous tributaries remodel so as to empty exclusively to the right side of the developing atrial component of the heart tube. Panels a through c show the remodelling taking place during Carnegie stages 12 through 14 that incorporates the left sinus horn into the developing left atrioventricular junction, with the vestibular spine, shown in purple, anchoring the primary atrial septum, shown in green, to the superior atrioventricular cushion. The dotted lines show the boundary between the systemic venous sinus and the remainder of the developing right atrium. The yellow structure is the developing sinus node. Panels d and e are sections taken from the annotated dataset for Carnegie stage 13 (specimen 11,559) and stage 14 (specimen A174). They confirm that the left sinus horn retains its own walls as it extends through the left atrioventricular junction.

As was shown in the reconstructions prepared for Carnegie stages 10 and 11 (Figures 2 and 3), the venous plexuses formed with the yolk sac, the placenta, and the embryo itself, were initially symmetrical. The vitelline and umbilical circulations come together first, forming the venous inflows to the developing heart tube. By Carnegie stage 11, they have been joined by the cardinal venous plexuses developed within the embryo. Subsequent to the union of the tributaries of the venous returns, the venous inflows can be recognised as the right and left horns of the developing systemic venous sinus. At Carnegie stage 12, the horns of the systemic venous sinus themselves still open in symmetrical fashion to the base of the developing atrial component of the heart tube (Figure 6a). With the development of anastomotic channels in the cranial part of the embryo itself, there is a shift of venous flows towards the right side. Concomitant with these changes, the left horn of the systemic venous sinus, retaining its own walls, becomes part of the developing left atrioventricular junction (Figure 5b). During these periods, the venous valves have formed within the right side of the initial atrial component of the heart tube, producing obvious boundaries between the cavities of the atrium and the systemic venous sinus (Figure 5d). The shift of the systemic venous sinus to the right side of the developing atrium then permits growth of the primary septum from the atrial roof, sequestrating the venous sinus to the right side (Figure 5c,d). By this time, the initial connection between the developing heart tube and the pharyngeal mesenchyme, which formed an extensive dorsal mesocardium, has largely disappeared. It persists, however, at the dorsal extent of the systemic venous sinus. This connection with the pharyngeal mesenchyme permits additional tissues to enter the heart as the vestibular spine (Figure 5e). This new material is key for committing the pulmonary vein to the developing left atrium.

## 6 FORMATION OF THE PULMONARY VEIN

**FIGURE 7:**
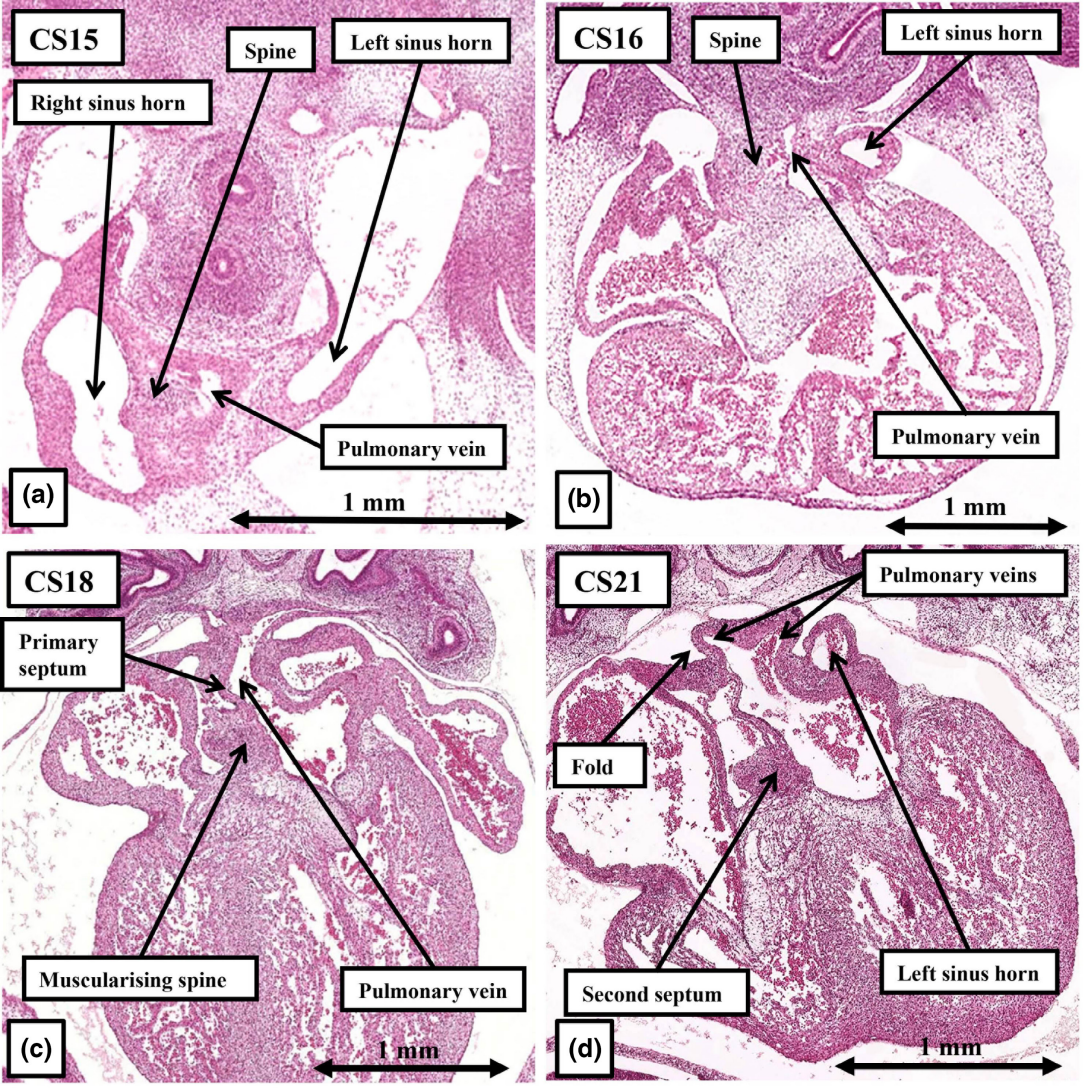
The sections, taken from the annotated datasets for Carnegie stages 15, 16, 18 and 21, show that the pulmonary vein does not canalise within the persisting dorsal mesocardium (a) until after the systemic venous sinus has remodelled so as to open exclusively to the developing right atrium (b). Initially, as seen at Carnegie stage 16 (b), the pulmonary vein opens through a solitary orifice at the level of the left atrioventricular junction (c). Not until Carnegie stage 21 (d) do the venous orifices reach the atrial roof, with this remodelling producing the superior interatrial fold. Still further remodelling in the foetal period is required before the four pulmonary veins open into the corners of the roof of the left atrium.

The origin of the pulmonary vein had been a contentious topic throughout the latter part of the twentieth century, with controversy continuing at the end of the opening decade of the 21st century (Douglas et al., 2011). By this time, nonetheless, molecular evidence had already shown that the pulmonary vein had no relationship to the tributaries of the systemic venous sinus (Sizarov et al., 2010). The sections available from the annotated datasets of the HDBR Atlas provide the morphological evidence that endorses the molecular approach. The pulmonary vein, as a perforate channel joining the pulmonary venous plexuses with the atrial cavity, is not seen until CS13–15. By this time, the systemic venous tributaries have already been sequestered to the cavity of the developing right atrium (Figure 6d). The pulmonary venous orifice is found within the pharyngeal mesenchyme of the dorsal mesocardium to the left of the developing vestibular spine (Figure 7a). It is then the growth of the spine into the atrial cavity that confines the solitary pulmonary venous orifice within the developing left atrium, initially at the level of the left atrioventricular junction (Figure 7b). This change is evident at CS16, by which time 6 weeks have passed subsequent to conception. By CS18, 1 week later, the venous orifice has begun to migrate cranially towards the atrial roof (Figure 7c). The spine, along with the mesenchymal cap carried on the leading edge of the primary atrial septum have also, by this stage, begun to myocardialise to form the secondary atrial septum (Figure 7c). By CS21, in the eighth week after conception, separate right and left venous orifices can be recognised opening to the roof of the left atrium (Figure 7d). It is only subsequent to the migration of the right pulmonary venous orifice to the roof of the left atrium that it becomes possible to recognise the superior interatrial fold, which is frequently described incorrectly as the secondary atrial septum. Only during foetal life, however, does ongoing remodelling result in four pulmonary veins achieving their definitive positions at the corners of the left atrial roof (Webb et al., 2001).

## 7 FORMATION OF THE ATRIAL SEPTUM

**FIGURE 8:**
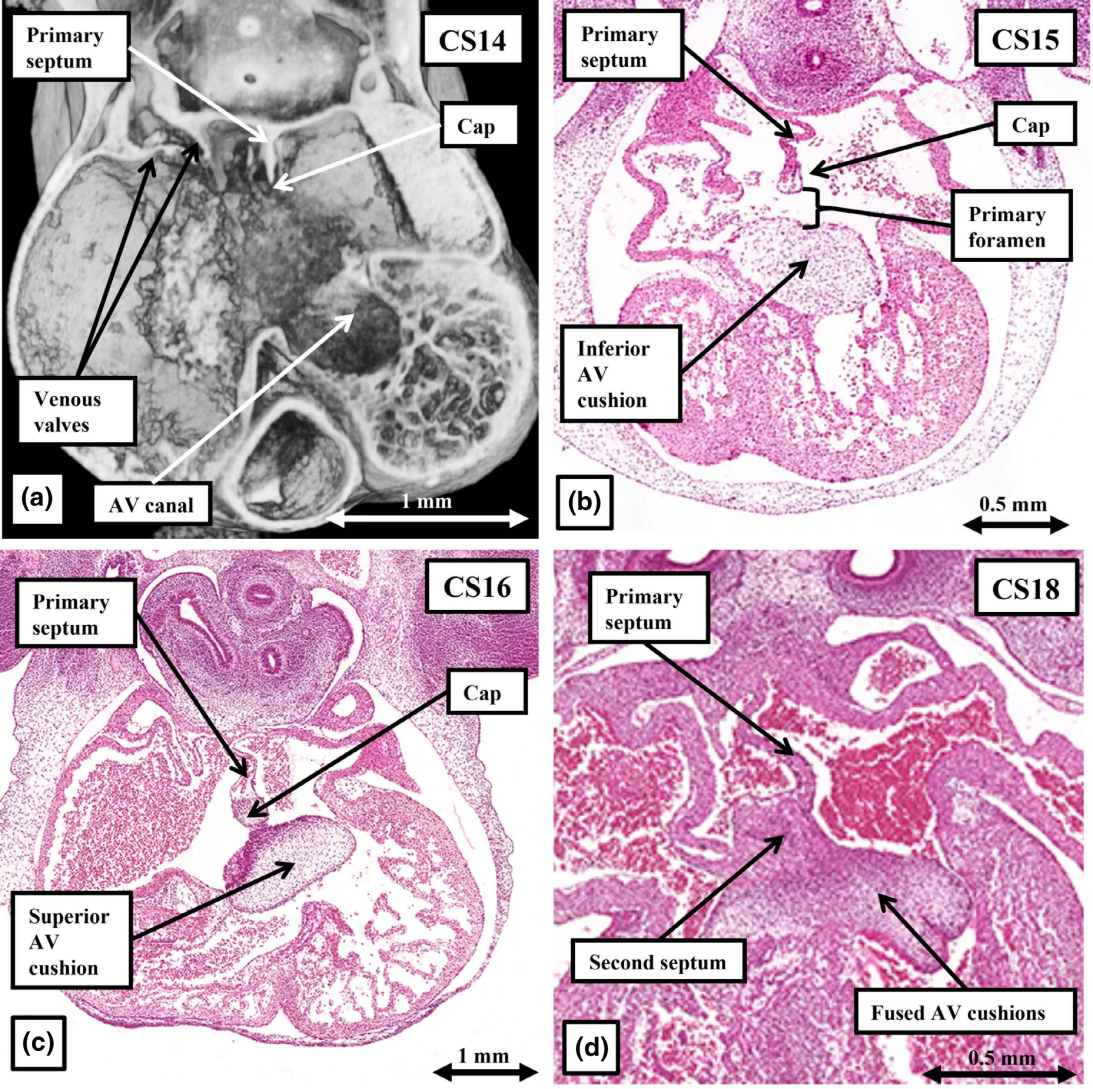
The images show the stage of atrial septation. Panel (a) is a section from an episcopic dataset prepared from an embryo at Carnegie stage 14. It shows the initial growth of the primary atrial septum to the left of the systemic venous sinus, which by this stage has remodelled such that it opens exclusively to the right side of the atrial component of the heart tube. Already at this initial stage the septum has a mesenchymal cap on its leading edge. By Carnegie stage 15 (b), the septum and its mesenchymal cap are growing s the atrioventricular cushions, which by now have separated the atrioventricular canal into the tricuspid and mitral valvar orifices. By Carnegie stage 16, the cap on the septum is fusing with the cushion mass to obliterate the primary atrial foramen (c). By Carnegie stage 18, the zone of fusion has been reinforced by growth of the rightward margin of the dorsal mesocardium, known as the vestibular spine (see Figure 6—upper right hand panel). The spine and mesenchymal cap then muscularise to form the second atrial septum (d).

**FIGURE 9:**
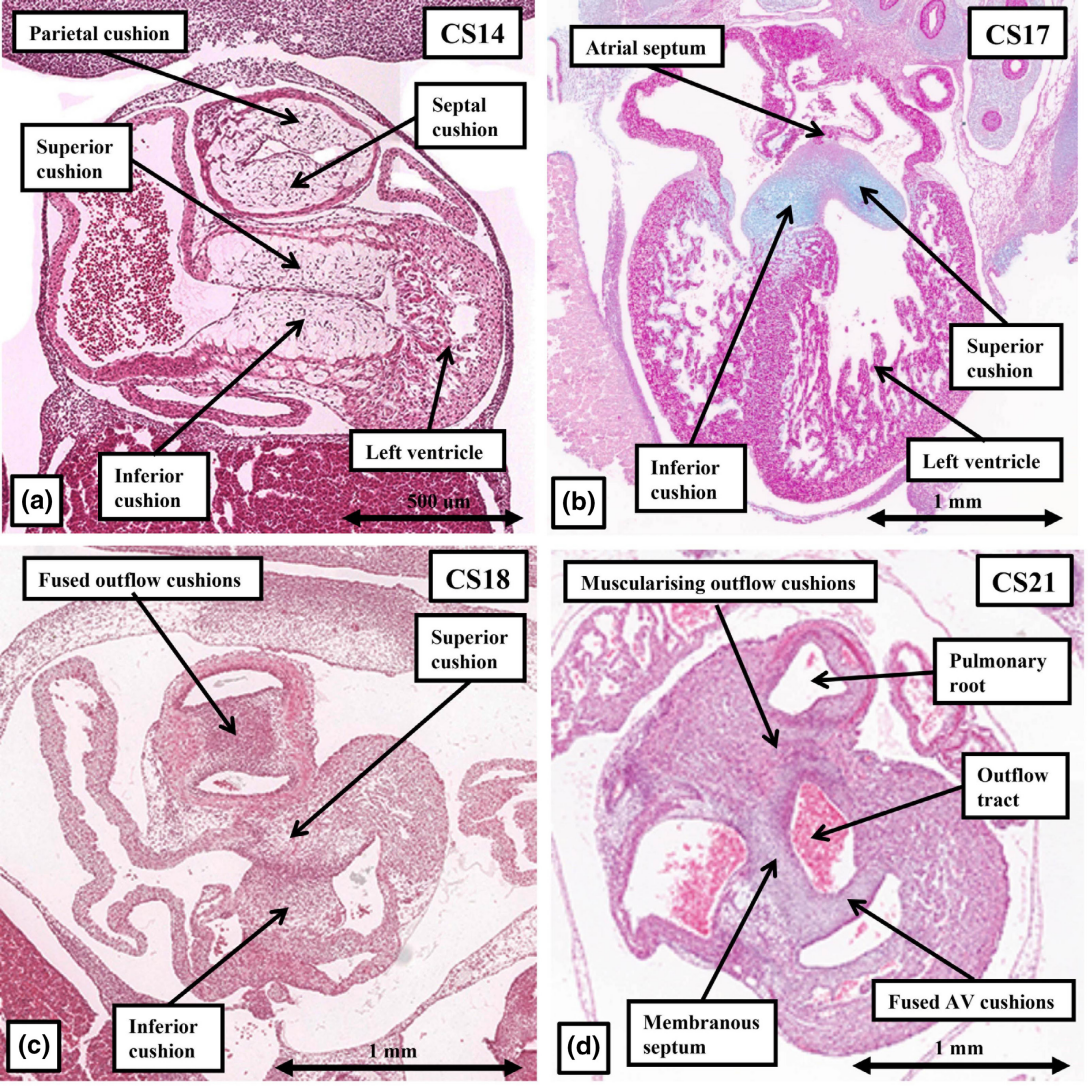
The sections show the fate of the atrioventricular cushions, by Carnegie stage 14 positioned superiorly and inferiorly within the atrioventricular canal (a). As shown in (b), by CS17 the larger parts of the atrioventricular cushions are positioned above the cavity of the developing left ventricle, providing a base for insertion of the atrial septum. By the next stage (c), the cushions have begun to fuse, dividing the canal into the tricuspid and mitral valvar orifices. The proximal outflow cushions can also be seen, at this stage, to have divided the outflow tract into the aortic and pulmonary channels. By Carnegie stage 21, the basal parts of the atrioventricular cushions have produced a bay within the base of the ventricular mass, which is now the dorsal border of the outflow tract, with the rightward margins of the cushions having fused to close the tertiary interventricular foramen (d).

The scene for atrial septation is set by the remodelling of the systemic venous tributaries, which by CS13 open exclusively to the right side of the atrial component of the initial heart tube. By this stage, the venous valves have formed, providing the boundary between the systemic venous sinus and the developing right atrium (Figure 6d). The atrial component of the heart tube, however, still retains its connection to the pharyngeal mesenchyme through the persisting part of the dorsal mesocardium (Figure 6e). It is the rightward shift of the systemic venous sinus that permits the primary atrial septum to grow from the atrial roof to its left side. From the time of its initial formation, the primary septum carries on its leading edge a mesenchymal cap (Figure 8a). The cap, formed by the process of endothelial‐to‐mesenchymal transformation, is continuous with the superior atrioventricular cushion. By Carnegie stage 15, at the beginning of the sixth week of development, the primary septum is growing towards the atrioventricular cushions (Figure 8b), with the cushions themselves now lying edge‐to‐edge within the atrioventricular canal, separating it into the tricuspid and mitral valvar orifices (Figure 9a). Growth of the septum, and its cap, towards the cushions reduces the size of the primary atrial foramen (Figure 8c). The primary septum by this time has broken away from the atrial roof, allowing the richly oxygenated umbilical venous return to continue to reach the left side of the developing heart, thus forming the secondary interatrial foramen. The site of fusion between the mesenchymal cap and the atrioventricular cushions is then reinforced by growth of the mesenchymal tissues of the vestibular spine, a process initially described by Wilhem His the Elder (Webb et al., 1998). It is myocardialisation of the vestibular spine and the mesenchymal cap that produces the true second atrial septum, which forms the antero‐inferior buttress of the oval fossa (Figure 8d; Jensen et al., 2017). The structure most frequently described as the “septum secundum” is no more than an infolding of the atrial roof between the right pulmonary veins and the intersepto‐valvar space of the right atrial roof (Rose, 1889). This fold does not become evident until the right pulmonary veins have remodelled such that they connect to the roof of the developing left atrium (Figure 7d). The process of remodelling will permit the upper margin of the primary atrial septum to overlap the fold. This arrangement then ensures closure of the oval foramen as soon as left atrial pressure exceeds the pressure in the right atrium in postnatal life. The primary atrial septum then forms the flap valve of the oval foramen, although anatomical fusion between the flap and the fold occurs in only around four‐fifths of individuals. Those without fusion have persistent patency of the oval foramen (Hagen et al., 1984).

## 8 FURTHER DEVELOPMENT OF THE ATRIOVENTRICULAR CANAL

As shown in Figure 5, the atrioventricular canal, subsequent to formation of the ventricular loop during the fifth week, is supported exclusively above the cavity of the developing left ventricle, whilst its rightward margin provided contiguity between the developing parietal walls of the right atrium and the right ventricle. It is this contiguity that, by the beginning of the fifth week of development, has permitted the canal to expand so as to provide the right ventricle with its own inlet (Figure 5b,d). By CS12, the endocardium lining the atrioventricular canal has undergone a process of endothelial‐to mesenchymal transformation resulting in migration of cells into the neighbouring cardiac jelly (Eisenberg & Markwald, 1995). It is this process that produces the atrioventricular endocardial cushions. When assessed in the short axis of the atrioventricular canal, at the beginning of the sixth week of development, the cushions lie edge‐to‐edge (Figure 9a). They separate the canal into the potential tricuspid and mitral valvar orifices. With the expansion of the atrioventricular canal, and the remodelling of the primary interventricular communication, the rightward margins of the cushions drape themselves across the crest of the dorsal part of the developing muscular ventricular septum, albeit with most of the fusing cushions lying within the cavity of the left ventricle (Figure 9b). By CS18, at the beginning of the seventh week of development, the edges of the cushions are beginning to fuse (Figure 9c). As was shown at CS17 (Figure 9b), the larger parts of the cushions have remained within the left ventricle. By CS21, at the beginning of the eighth week, their left ventricular components have formed what will become the aortic leaflet of the mitral valve (Figure 9d). The smaller part of the inferior cushion, by this stage, remains draped across the dorsal part of the ventricular septum, with the fused cushions forming the dorsal margin of the secondary interventricular foramen. As we will describe, it is then fusion of extensions of the rightward margins of the two cushions, described by Odgers as the “tubercles” (Odgers, 1938) that closes the persisting aorto‐right ventricular communication, itself by then forming the tertiary interventricular communication (Figure 9d).

## 9 REMODELLING OF THE PHARYNGEAL ARCHES

**FIGURE 10:**
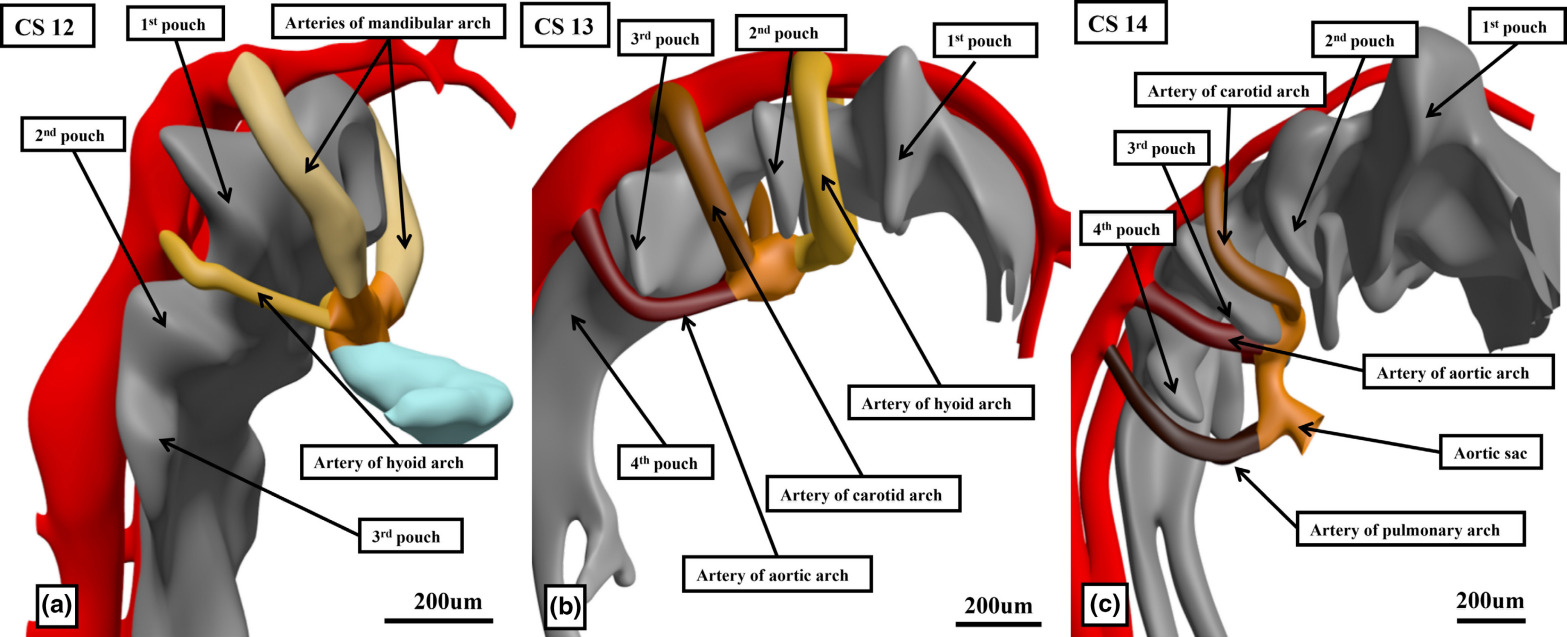
The images, prepared from the interactive PDFs show the remodelling that takes place in the pharyngeal mesenchyme, with the concomitant changes that occur with remodelling of the arterial channels that form within the pharyngeal arches, themselves demarcated by formation of the pharyngeal pouches. At Carnegie stage 12 (a), a manifold has developed within the ventral pharyngeal mesenchyme that is known as the aortic sac. At this initial stage, as shown in the left hand panel, the manifold gives rise to arteries that percolate in symmetrical fashion through the first two pharyngeal arches. As shown in the middle panel, by Carnegie stage 13 (b), there has been formation of the two more pharyngeal arches, but with resorption of the arteries that extended through the first arch. By Carnegie stage 14 (c), an additional and ultimate pharyngeal arch has formed, but now with resorption of the larger part of the arteries of the second arch. This means that only five pharyngeal arches are formed during human development, creating problems for those who describe formation of a sixth arch, postulating vestigial formation of the fifth arch. To circumvent these problems in nomenclature, it is preferable to name the arches, rather than numbering them, recognising carotid, aortic and pulmonary arches and arteries. It is the artery of the left pulmonary arch that becomes the arterial duct.

During the changes that have taken place at the venous pole of the heart to produce the separated systemic and pulmonary venous returns, equally important changes have been occurring at the arterial pole (Graham et al., 2023). As was seen at CS10, two arterial channels extended from the arterial pole, passing to either side of the developing gut to become the dorsal aortas. The extension laterally of the endoderm of the gut then produces pouches, which separate the pharyngeal mesenchyme into the pharyngeal arches. Over this period, a manifold, known as the aortic sac, is produced within the ventral pharyngeal mesenchyme. This is confluent at the margins of the pericardial cavity with the solitary cavity of the outflow tract. It is the aortic sac that then gives rise to the arteries forming within the pharyngeal arches. The arteries themselves initially extend in symmetrical fashion through the pharyngeal arches, joining posteriorly to form the dorsal aortas. By the end of CS12, during the fifth week after conception, two arches with arteries have formed (Figure 10a). An additional set of arches and arteries is formed by CS13. Already by this time, the arteries initially extending through the first set of arches has effectively disappeared (Figure 10b). By CS14, at the end of the fifth week after conception, the full set of arches has been formed (Figure 10c). Contrary to conventional wisdom, there are only five pairs of arches formed, and hence only five pairs of arteries. The numbering of the pharyngeal arches and their arteries from one to six, presuming the presence of a vestigial fifth arch, can be traced back to Boas, although he provided no evidence to substantiate his speculations (discussed in Anderson, Graham, et al., 2023). The wealth of literature that interprets congenital malformations on the basis of the non‐existent vestigial fifth arch makes it difficult to correct the initial mistake of Boas simply by correctly numbering the arches as one through five. It is preferable, therefore, to name the arches, and their respective arteries, rather than numbering them. The fourth arch can be named the aortic arch, whilst the ultimate arch becomes the pulmonary arch (Graham et al., 2023; Figure 10c). This then permits the arterial duct to be recognised as the persisting artery of the left pulmonary arch.

## 10 DEVELOPMENT OF THE DISTAL OUTFLOW TRACT

**FIGURE 11:**
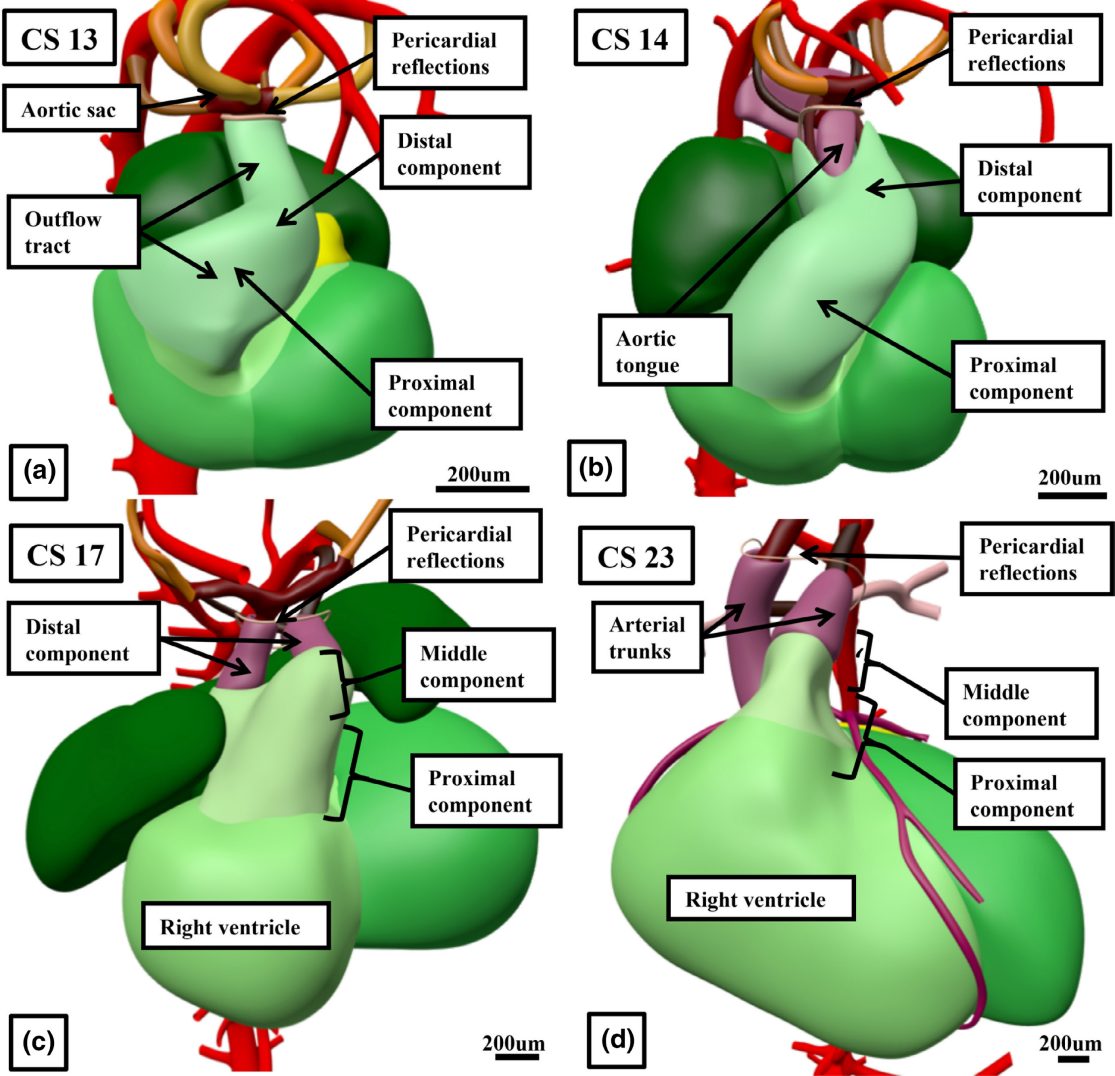
The reconstructions (a–d), made from the interactive PDFs show the increasing “regression” of the distal myocardial border that occurs as the migration of non‐myocardial cells from the heart‐forming areas into the arterial pole of the heart tube produces the intrapericardial arterial trunks.

**FIGURE 12:**
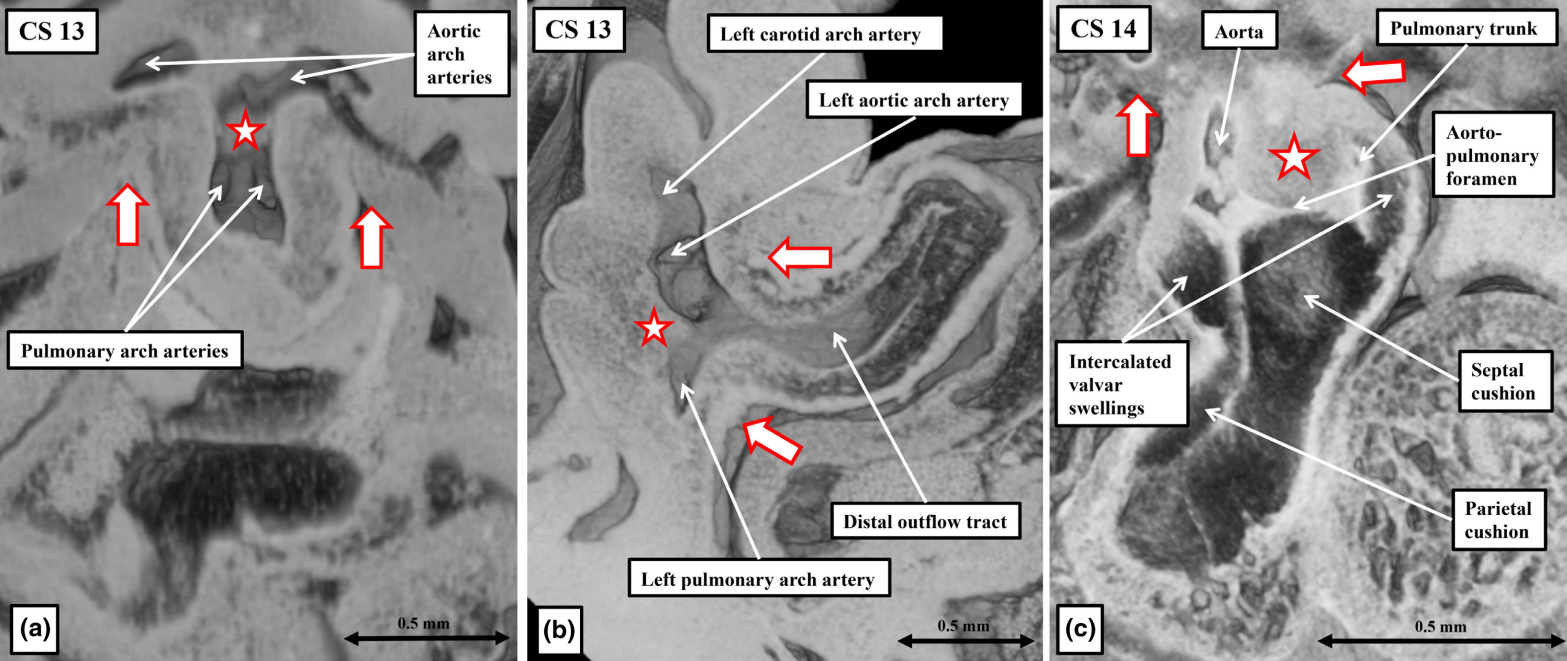
The sections are made using episcopic datasets prepared from different human embryos at Carnegie stage 13 (panels a and b) and stage 14 (panel c). Panels a and b show the junction between the distal outflow tract and the aortic sac as seen in frontal (panel a) and sagittal (panel b) fashion. The white star with red borders shows the dorsal wall of the aortic sac, which will protrude between the origins of the systemic and pulmonary arch arteries to become the aortopulmonary septum, as seen in panel c. The white arrows with red borders show the reflections marking the margins of the pericardial cavity. Panel c, which is another frontal section, shows the aortopulmonary foramen that exists until the aortopulmonary septum fuses with the distal margins of the major outflow cushions, which divides the part of the outflow tract that retains its myocardial walls into the aortic and pulmonary channels. The section also shows the intercalated valvar swellings, which will excavate, along with the distal margins of the major outflow cushions, to form the leaflets of the arterial valves (see Figure 13).

When first recognised, towards the end of the fourth week of development, the outflow tract extends from the developing right ventricle to the margins of the pericardial cavity. It initially has exclusively myocardial outer walls, with a solitary lumen lined with endocardial cells with interposed cardiac jelly. It is markedly tortuous at this initial stage, with an obvious dog‐leg bend permitting distinction, at CS13, of proximal and distal components (Figure 11a). By the beginning of the fifth week of development, there has been ongoing movement of cells from the second heart field into the arterial pole of the heart tube. These new cells do not immediately differentiate, however, and will not form myocardium. They initially form parietal tongues to produce a fishmouth configuration to the distal outflow tract (Figure 11b; Anderson et al., 2012). At the initial stage, when the outflow tract had exclusively myocardial walls, the remodelling of the aortic sac had produced wide separation between its cranial part, which gives rise to the arteries of the carotid and aortic arches, and the caudal part, which gives rise to the arteries of the pulmonary arch (Figure 12a). Already at this stage, the start of the fifth week of development, the dorsal wall of the sac is beginning to protrude ventrally between the origins of the systemic and the pulmonary arteries (Figure 12b). By the sixth week, the second heart field cells in the distal outflow wall are differentiating to become smooth muscle cells. They have extended sufficiently into the walls of the distal outflow tract to change the fishmouth configuration to an arrangement with an annular distal myocardial border (Figure 11c). The protrusion from the dorsal wall of the aortic sac has itself by then grown into the cavity of the distal outflow tract as an aortopulmonary septum (Figure 12c). The aortopulmonary septum then fuses with the distal ends of the main outflow cushions, which themselves also fuse. During the same period, the proximal margins of the parietal tongues of non‐differentiated tissue in the outflow walls are expanding and differentiating to form the intercalated valve swellings (Figure 12c). These structures, first described by Kramer, interdigitate with the margins of the major outflow cushions, thus producing the primordiums of the arterial roots (Eley et al., 2018; Kramer, 1942).

## 11 THE DEVELOPMENT OF THE ARTERIAL ROOTS

**FIGURE 13:**
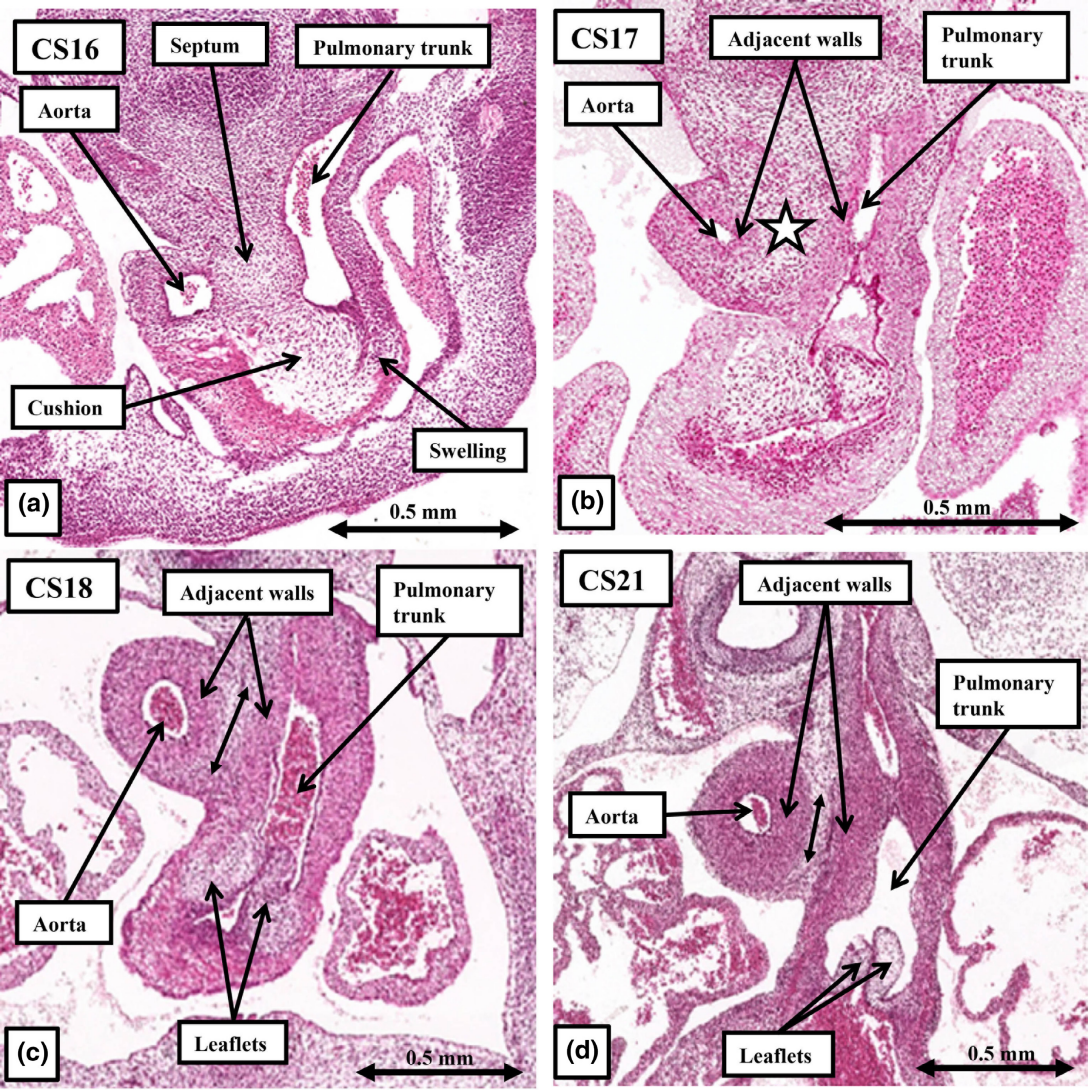
The images show the progressive remodelling of the distal margins of the outflow cushions, along with the intercalated valvar swellings, within the middle part of the outflow tract to produce the leaflets of the pulmonary valve. The remodelling commences whilst the cushions remain encased in the middle part of the outflow tract that has retained its myocardial walls. The ongoing addition of non‐myocardial tissues from the heart‐forming areas, however, produces the pulmonary valvar sinuses (a–d). A similar process of remodelling produces the leaflets of the aortic root. Note also that during the process of remodelling, the intrapericardial arterial trunks develop their own individual walls, with the intervening mesenchyme (star in panel b) losing its “septal” function (double‐headed arrows in panels c and d).

In addition to describing the intercalated valve swellings, Kramer also divided the outflow region into the “truncus” and the “conus.” We describe the outflow tract in three parts and take the “truncus” of Kramer to represent the component that will become the intrapericardial arterial trunks. The walls of these trunks are formed from cells originating in the second heart field and the neural crest. They will, in time, differentiate directly to become smooth muscle and fibrous tissue. As described, the cavity of the newly formed non‐myocardial distal outflow tract is partitioned by the growth of the aortopulmonary septum as a protrusion from the dorsal wall of the aortic sac. When first formed (Figure 12c), the protrusion is a true septal entity, separating the developing cavities of the intrapericardial aorta and pulmonary trunk. During the sixth week of development, at CS16, it still retains its septal identity (Figure 13a). By this stage, the distal parts of the major outflow cushions have themselves fused, separating what is now the middle part of the outflow tract into the developing pulmonary and aortic roots. In each root, the distal margins of the main cushions, which can now be seen as distinct from the proximal parts which are involved in outflow tract septation, will begin to cavitate. A similar process occurs in the intercalated valve swellings (Figure 13b). As the cushions and swellings cavitate to form the pockets of the sinuses during the eighth week after conception, so the walls of the arterial roots that encase them remodel. By the time the sinuses have formed, the most proximal part of the wall remains myocardial, but the distal part is arterial (Figure 13c,d). The sinuses themselves, however, require myocardial support as they, too, separate from one another. The support is provided by muscularisation of the distal margins of the major cushions (Anderson, Lamers, et al., 2023).

## 12 DEVELOPMENT OF THE PROXIMAL OUTFLOW TRACT

**FIGURE 14:**
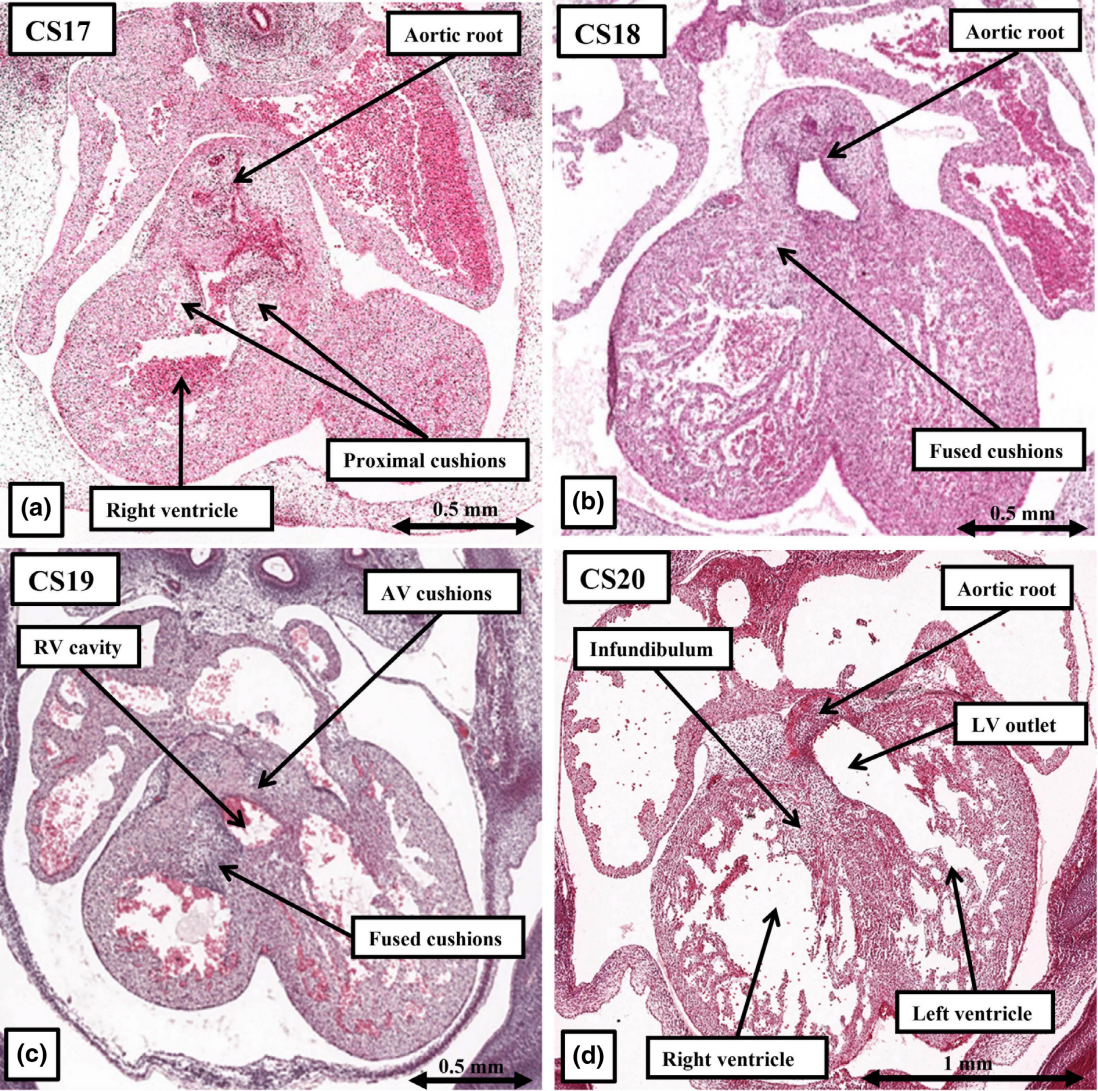
The images show the remodelling of the proximal parts of the outflow cushions, which fuse and muscularise from CS17–20 (a–d), initially forming a shelf in the roof of the right ventricle to commit the aortic root to the left ventricle, with the shelf itself becoming the free‐standing subpulmonary infundibular sleeve.

**FIGURE 15:**
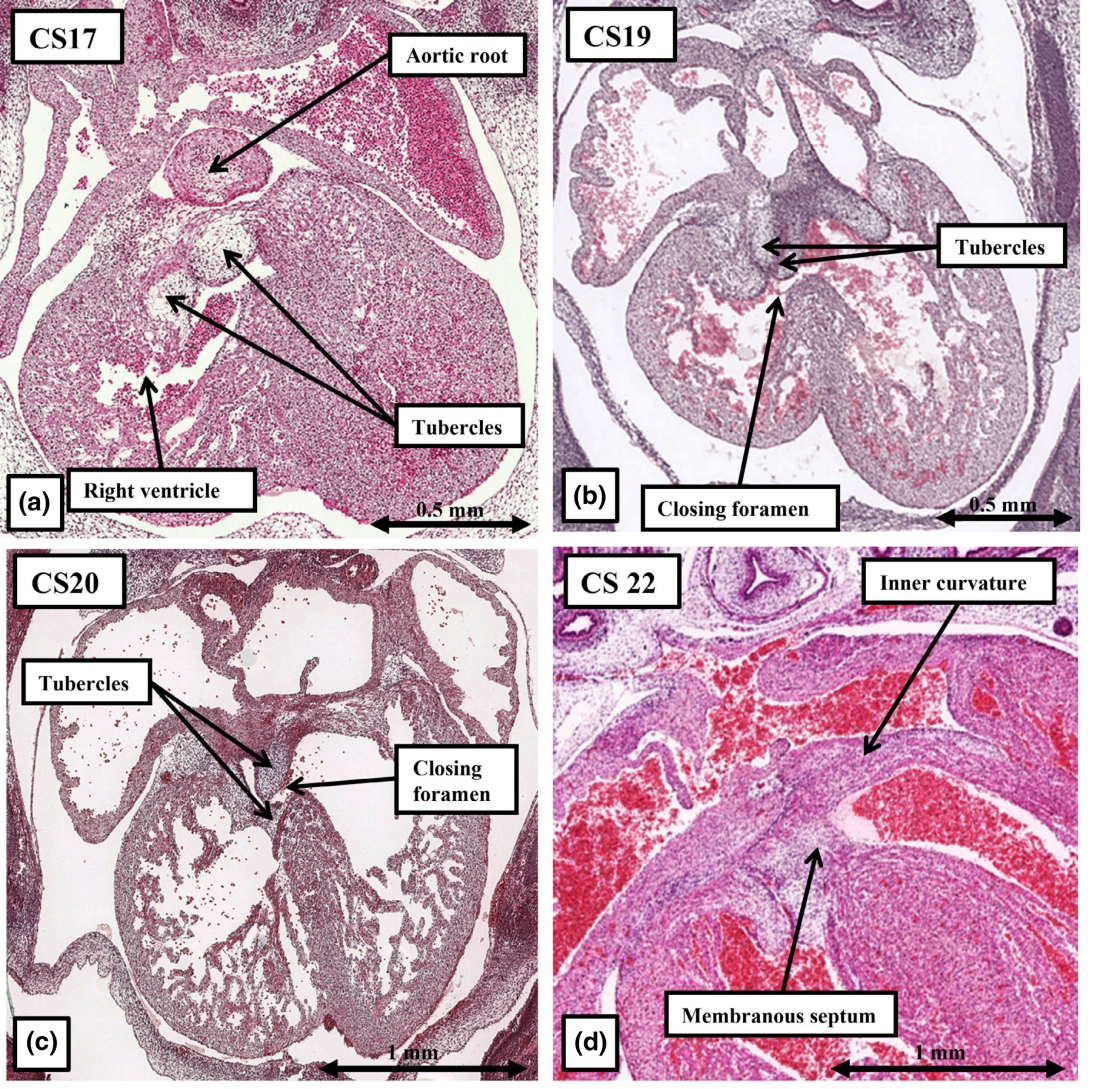
The images show how, subsequent to the formation of the shelf in the roof of the right ventricle by fusion and muscularisation of the proximal parts of the outflow cushions, the rightward margins of the atrioventricular cushions, known as the tubercles, fuse with each other and then with the proximal outflow cushions to close the aorto‐right ventricular communication that represents the tertiary interventricular communication (a–c). As is shown in panel d, by Carnegie stage 22 the tubercles have become the membranous part of the ventricular septum.

During the sixth week of development, there has been fusion, in distal to proximal direction, of the outflow cushions that separate the outflow tract into its aortic and pulmonary channels. Thus, towards the end of the sixth week of development, at CS17, the most proximal parts of the outflow cushions have yet to fuse (Figure 14a). Even as fusion extends to involve the proximal cushions, the aortic root retains its location above the cavity of the right ventricle (Figure 14b). Throughout this period, therefore, the exit from the left ventricle to the aortic root is the secondary interventricular communication (Figure 14c). By the middle of the seventh week of development, at CS19, the proximal cushions have also begun to myocardialise, thus building a shelf beneath the aortic root. This process creates a tunnel that places the root, through the secondary interventricular communication, into continuity with the cavity of the left ventricle (Figure 14c). Whilst the tunnel is being built, a communication remains between the aortic root and the cavity of the right ventricle. This aorto‐right ventricular communication is then effectively a tertiary interventricular communication. This is because, by this time, the space beneath the aortic root, although initially belonging to the right ventricle, is becoming the left ventricular outflow tract (Figure 14d). With closure of the tertiary foramen (Figure 15), the muscular shelf separates the cavity of the right ventricle from the newly formed aortic root. It then becomes the free‐standing right ventricular infundibular sleeve (Figure 14d).

## 13 CLOSURE OF THE TERTIARY INTERVENTRICULAR FORAMEN

As was explained above, the fusion and myocardialisation of the proximal outflow cushions created a shelf at the base of the right ventricle. This process then placed the aortic root in communication with the cavity of the left ventricle (Figure 14c). But, a communication still remained between the aortic root and the cavity of the right ventricle. This was the tertiary interventricular foramen. Its caudal border is made up of the rightward margins of the atrioventricular cushions, which are the “tubercles” (Odgers, 1938; Figure 15a,b). The foramen is bounded cranially by the leading edge of the fused and myocardialised outflow cushions. The atrioventricular cushions, in particular the inferior cushion, will also contribute to the formation of the septal leaflet of the tricuspid valve (Figure 15c). By the end of the seventh week of development, at around CS20, the tubercles have fused with each other. Shortly thereafter, they have also fused with the crest of the ventricular septum and the proximal outflow cushions to obliterate the aorto‐right ventricular communication (Figure 15d). With ongoing development, they become the membranous part of the ventricular septum. It is only subsequent to delamination of the septal leaflet of the tricuspid valve, which takes place during the foetal period of development, that the membranous septum becomes separated into its interventricular and atrioventricular components (Allwork & Anderson, 1979). With regard to the formation of the ventricular septum itself, it has only the muscular part, formed concomitant with ballooning of the apical trabecular components, and the membranous part formed from the tubercles of the atrioventricular cushions. In the normal heart, there is no discrete “outlet septum.”

## 14 DEVELOPMENT OF THE VENTRICULAR WALLS

**FIGURE 16:**
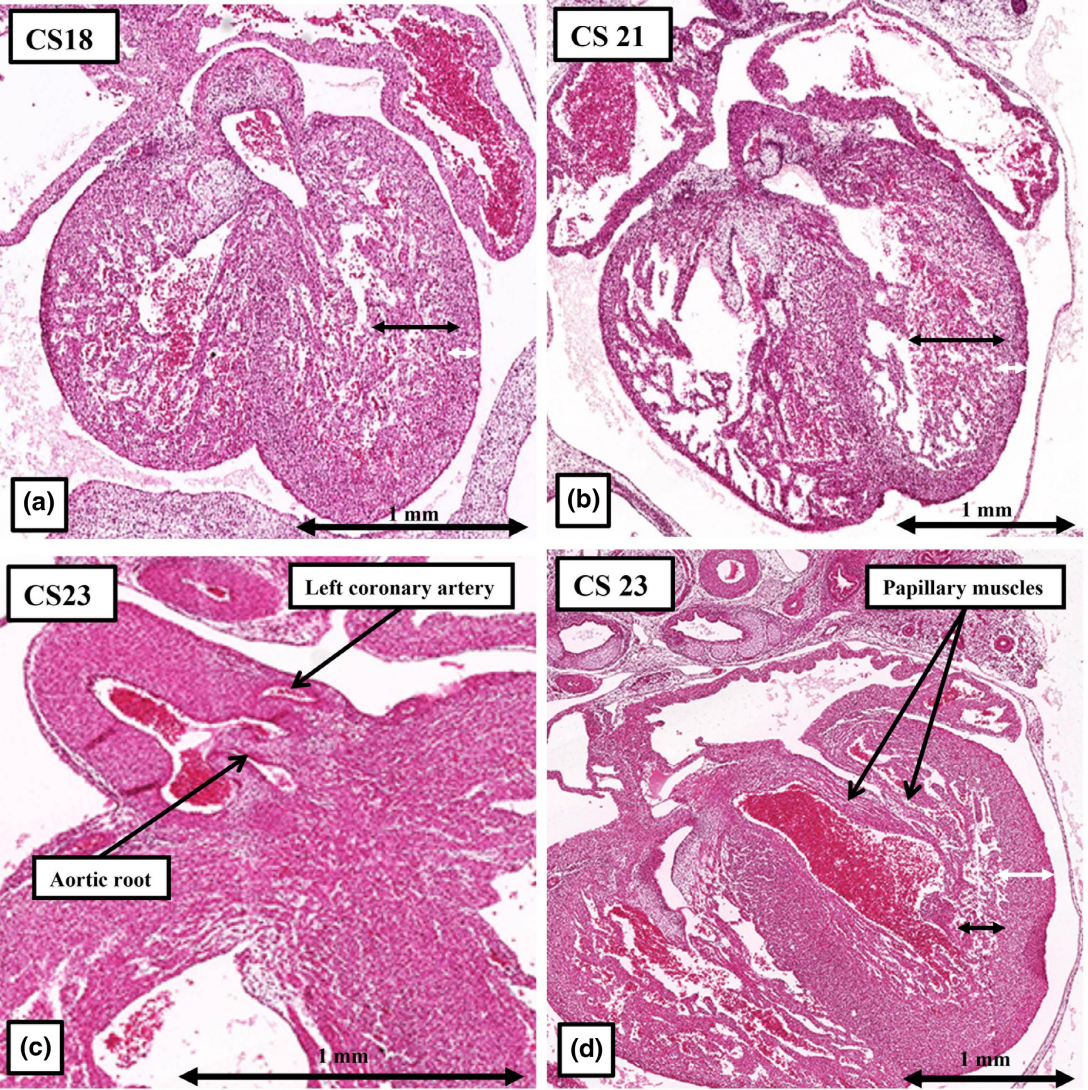
The images show how, subsequent to the connection of the epicardial coronary arteries to the aortic root, the walls of the ventricular chambers change from being mostly made up of a meshwork of trabeculations (double headed black arrows) to becoming predominantly compact (double headed white arrows in a, b). The connection between the aortic root and the epicardial coronary arteries is achieved through the growth of stems from the developing aortic valvar sinuses, as shown in panel c. The initial trabecular layer, as shown in the panel d, then coalesces to become the papillary muscles of the atrioventricular valves, and the ventricular ramifications of the conduction system. There is no evidence to show that the trabeculations coalesce to form the compact ventricular wall.

The literature is replete with accounts of so‐called “ventricular non‐compaction.” This description is based on the notion that the trabeculations, which initially form the larger parts of the thickness of the ventricular walls, coalesce to become compact, and hence form the compact components of the walls. There is no evidence to support this notion (Petersen et al., 2023). It is true that the thickness of the ventricular walls, at the beginning of the seventh week of development, is made up largely of trabeculations (Figure 16a). At this stage, however, although the coronary arteries have begun to develop within the epicardial layers, they have no communication with the aortic root. It is the establishment of the continuity between the aortic root and the epicardial coronary arterial network (Figure 16c) that correlates with the rapid growth of the compact myocardial layer (compare Figure 15b,d). But the growth of the compact later reflects the rapid proliferation of the compact cardiomyocytes, rather than coalescence of pre‐existing trabeculations. The trabeculations, nonetheless, do coalesce to form that papillary muscles of the atrioventricular valves (Figure 15d). They also form the ramifications of the ventricular conduction system. The mechanism of connection of the epicardial coronary arteries with the aortic root has also been controversial. Evidence is emerging to suggest that short stems grow out from the developing aortic valvar sinuses to join an extensive epicardial plexus that eventually remodel to form the mature coronary network (Anderson et al., 2022).

## 15 CONCLUSIONS

In this account we have discussed only the stages of embryonic development. Significant changes in the human heart continue during the foetal period, in particular the maturation and modelling of the cardiac valves. It is also not until the foetal period of development that it becomes possible to recognise the inferior pyramidal space and the infero‐septal recess of the left ventricle (Tretter et al., 2022). As indicated, foetal datasets are being added to the resource. The availability of these datasets will permit clarification of the steps involved in the formation of these relatively unrecognised components of the normal heart. The purpose of our review, nonetheless, was to show how the evidence now available from interrogation of the materials making up the HDBR Atlas makes it possible for the interested observer to arbitrate for themselves the ongoing controversies that continue to plague the understanding of cardiac development. The HDBR atlas, furthermore, remains a work in progress. As more material is added to its component parts, such as datasets covering the foetal period of development, the provision of sections showing the specific location of genes, and differentiation of the myocardial components of the developing heart, the information available will become increasingly robust. Moreover, we are currently working towards a Gene Expression Portal that will not only enhance the usefulness of the dataset by adding the much needed, and currently lacking, ability to search and query the data. This will also provide the ability to link out from the gene expression section to the nearest annotated anatomical section on the HDBR eHistology viewer—thus, providing a tool to enable molecular data to be directly compared with the underlying tissue histology. Thanks to the production of resources such as that devoted to Human Development, no longer is there any justification for suggesting that embryology is a hindrance rather than a help.

## Data Availability

Data sharing is not applicable to this article as no new data were created or analyzed in this study.
